# HIV signaling through CD4 and CCR5 activates Rho family GTPases that are required for optimal infection of primary CD4+ T cells

**DOI:** 10.1186/s12977-017-0328-7

**Published:** 2017-01-24

**Authors:** Mark B. Lucera, Zach Fleissner, Caroline O. Tabler, Daniela M. Schlatzer, Zach Troyer, John C. Tilton

**Affiliations:** 1 Division of Infectious Diseases, Anschutz Medical Campus, University of Colorado, Aurora, CO USA; 2 Department of Nutrition, Center for Proteomics and Bioinformatics, School of Medicine, Case Western Reserve University, 10900 Euclid Ave, BRB 919, Cleveland, OH 44106 USA

**Keywords:** HIV-1, Small molecule screen, Rho GTPases, ROCK, Cdc42, Viral infection

## Abstract

**Background:**

HIV-1 hijacks host cell machinery to ensure successful replication, including cytoskeletal components for intracellular trafficking, nucleoproteins for pre-integration complex import, and the ESCRT pathway for assembly and budding. It is widely appreciated that cellular post-translational modifications (PTMs) regulate protein activity within cells; however, little is known about how PTMs influence HIV replication. Previously, we reported that blocking deacetylation of tubulin using histone deacetylase inhibitors promoted the kinetics and efficiency of early post-entry viral events. To uncover additional PTMs that modulate entry and early post-entry stages in HIV infection, we employed a flow cytometric approach to assess a panel of small molecule inhibitors on viral fusion and LTR promoter-driven gene expression.

**Results:**

While viral fusion was not significantly affected, early post-entry viral events were modulated by drugs targeting multiple processes including histone deacetylation, methylation, and bromodomain inhibition. Most notably, we observed that inhibitors of the Rho GTPase family of cytoskeletal regulators—including RhoA, Cdc42, and Rho-associated kinase signaling pathways—significantly reduced viral infection. Using phosphoproteomics and a biochemical GTPase activation assay, we found that virion-induced signaling via CD4 and CCR5 activated Rho family GTPases including Rac1 and Cdc42 and led to widespread modification of GTPase signaling-associated factors.

**Conclusions:**

Together, these data demonstrate that HIV signaling activates members of the Rho GTPase family of cytoskeletal regulators that are required for optimal HIV infection of primary CD4+ T cells.

**Electronic supplementary material:**

The online version of this article (doi:10.1186/s12977-017-0328-7) contains supplementary material, which is available to authorized users.

## Background

HIV-1, like other viruses, relies on host cellular machinery to successfully complete replication. The proteins co-opted by HIV, collectively known as host dependency factors, have been investigated using a variety of approaches including functional genomic screens with siRNAs [1–3]. These studies have revealed that host dependency factors fall into a variety of pathways including cytoskeletal and transport proteins, components of nuclear pore complexes, LEDGF/p75, mRNA transcription, RNA export and translation machinery, ER/Golgi trafficking, and members of the ESCRT machinery [1, 2, 4]. Although these high-throughput screens have been informative in identifying novel host dependency factors, they rely on changes in HIV replication due to altered protein expression levels. However, it is well-recognized that protein function is controlled not only by expression levels, but also by a variety of post-translational modifications (PTMs) that may be overlooked in conventional knockdown screening approaches. For example, the histone acetyltransferases p300/CBP were not identified as host dependency factors in siRNA screens despite known interactions with Tat and integrase and reports of reduced HIV replication in the presence of specific p300 inhibitors [5–9].

In the context of HIV-1, PTMs play important roles in several steps of viral replication. Acetylation and methylation of histone residues on viral-associated nucleosomes impact chromatin structure and accessibility of the viral promoter to cellular transcriptional machinery [10, 11]. For instance, histone deacetylase (HDAC) inhibitors promote viral reactivation from latency both in vitro and in vivo and are being investigated as potential latency reversing agents in strategies to eliminate latent viral reservoirs [12–15]. Recently, we reported an unanticipated effect of HDAC inhibitors on HIV infection that was independent of its effects on nuclear histones: pre-treatment of primary CD4+ T cells with drugs that inhibited the cytoplasmic HDAC6 enhanced the kinetics and efficiency of reverse transcription, nuclear import, and integration and led to a significant increase in susceptibility to HIV infection [16]. The mechanism of this phenomenon, informed also by studies from Naghavi and colleagues [17–19], appears to be related to the formation of stable acetylated microtubule networks induced by HIV—and enhanced by blocking tubulin deacetylation by HDAC6. These networks subsequently serve as conduits for efficient migration of viruses from the cytoplasm to the nucleus [18–20].

Additionally, phosphorylation has also been implicated in regulating susceptibility of cells to HIV. A comprehensive phosphoproteomic analysis of signaling events induced by HIV binding to cell surface receptors via CD4 and CXCR4 revealed more than 200 phosphorylation sites that were altered following HIV exposure, several of which were confirmed to directly impact viral replication [21]. More recently, phosphorylation of the CDK9 T-loop (Thr-175) of the positive transcription elongation factor, P-TEFb, and of HEXIM1 have also been implicated in enhancing proviral HIV gene expression [22, 23]. Importantly, all of these post-translational modifications impacting HIV infection would be difficult or impossible to detect using conventional knockdown approaches.

In this study, we sought to investigate the role of PTMs on the efficiency of HIV-1 fusion and infection. To achieve this, we selected a commercially available panel of small molecule inhibitors tailored to enzymes involved in epigenetic and PTM pathways and analyzed them using a combination reporter virus system that measures both viral fusion and LTR-driven EGFP expression, which requires not only fusion but also post-entry steps including uncoating, reverse transcription, nuclear import, integration, Tat-dependent transcription and Rev-dependent mRNA export, and translation to occur successfully [24]. While the majority of compounds had no effect at the level of fusion, 39 compounds yielded greater than 0.5 log_2_ fold change in viral infection as measured by LTR-driven EGFP expression. Most notably, all histone deacetylase inhibitors tested increased EGFP expression, in agreement with our previous findings that pan-HDAC inhibitors vorinostat, panibinostat, romidepsin, and the HDAC6-specific inhibitor tubacin enhanced viral infection [16]. Conversely, inhibitors of histone and DNA methylation predominated the list of compounds that reduced HIV infection. Interestingly, the small molecule CCG-100602, reported to inhibit Rho GTPase signaling [25], was identified in our screen as a negative regulator of HIV. Based on this observation, we tested additional inhibitors of the Rho family GTPases Cdc42 and the Rho-associated protein kinase (ROCK) and observed similar reductions in HIV infection. Finally, using phosphoproteomic and biochemical analysis of signaling pathways altered by HIV binding to CD4 and CCR5, we observed that HIV signaling activates the small GTPases Rac1 and Cdc42 and induces a dramatic restructuring in the phosphorylation cascades of Rho family GTPases and associated proteins, suggesting that the activity of these pathways is modulated directly by the virus. Together, these data indicate that HIV signaling via its receptors at the time of attachment activates Rho family GTPases and that RhoA, Cdc42, and ROCK are host dependency factors for optimal HIV infection of primary CD4+ T cells.

## Methods

### Small molecule inhibitors

The 96-well “epigenetic screening library” was acquired from Cayman Chemical and stored at −20 °C as 10 mM stocks in DMSO (Batch #0449323). Small molecule inhibitors were serially diluted in RPMI 1640/10% FBS/1% PenStrep for a final effective range of 10 nM–10 μM immediately prior to experimental setup. Multiple freeze-thaw cycles were prevented to exclude potential variations in drug potency.

### Infection experiments

1 × 10^6^ unstimulated primary CD4+ T cells were plated per well in a 96-well format and incubated for 4 h at 37 °C with media alone or media supplemented with 10 nM, 100 nM, 1 μM, or 10 μM of indicated small molecule inhibitor. Parallel plates were prepared for analysis of viral fusion and LTR-driven EGFP expression as previously described [24]. Following incubation with small molecule inhibitors, cells were infected with HIV-1 reporter virus strain NL4-3-deltaE-EGFP (obtained through the NIH AIDS Research and Reference Reagent Program, Division of AIDS, NIAID, NIH: pNL4-3-delta-E-EGFP (Cat #11100) from Drs. Haili Zhang, Yan Zhou, and Robert Siliciano) bearing CXCR4-tropic envelope JOTO.TA1.2247 [26] and a β-lactamase-Vpr fusion protein [27, 28]. Plates were spinoculated for 2 h at 1200 rpm and 25 °C. Following centrifugation, cells were incubated for 1 h at 37 °C. Cells for viral fusion analysis were treated with CCF2-AM, washed, and incubated overnight at room temperature in the presence of probenecid. For EGFP expression, cells were incubated at 37 °C for a total of 72 h prior to staining and processing.

### Flow cytometry

Cells were washed once with PBS containing 1% BSA and incubated with live/dead near-IR fixable viability dye (Invitrogen), CD3 Brilliant Violet 650 (BioLegend), and CD4 Allophycocyanin (eBioscience) at 4 °C for 30 min. Cells were washed in PBS/BSA and fixed in PBS/BSA containing 1% paraformaldehyde prior to data acquisition. All samples were analyzed using a BD Fortessa (BD Biosciences) cell analyzer equipped with a High Throughput Sampler option. A minimum of 50,000 events were collected per sample at a flow rate of 2.5 μL/s with 50 μL mixing and 3 × 200 μL washes. All experimental conditions were performed in triplicate. FlowJo version 9.7.6 (TreeStar, Inc) was used for analysis. For downstream validation of significant hits, the above experiments above were repeated in triplicate on a minimum of 3 healthy donor CD4+ T cell populations.

### β-lactamase-mediated cleavage of CCF2 in HEK293T cells

Human embryonic kidney 293T/17 cells were seeded in 10 cm^2^ dishes at a density of 3 × 10^6^ cells per dish. Cells were transfected with 7.5 μg β-lactamase-Vpr plasmid using previously established calcium phosphate methods and fresh media was added 6 h post transfection. At 48 h post transfection, cells were incubated with media alone or media containing 10 μM chaetocin for 4 h. Cells were subsequently treated with CCF2-AM for 2 h at room temperature, washed, and incubated overnight in CO_2_-independent media containing probenecid. The following day, cells were trypsinized, stained with live/dead near-IR viability dye and analyzed for evidence of substrate cleavage.

### Phosphoproteomic analysis of HIV-exposed CD4+ T cells

Memory CD4+ T cells were purified by negative selection from a leukapheresis pack using custom RosetteSep kits (STEMCELL Technologies). Briefly, equal populations of 150 × 10^6^ cells were exposed to 20 μg/ml p24 equivalent AT2-inactivated HIV-1 THRO or protein equivalent concentrations of non-viral microvesicles as control. HIV-1 THRO is a transmitted/founder virus isolated from a subject with early HIV infection that was found to be CCR5-tropic as evidenced by inhibition by TAK779 but not AMD3100 on TZM-bl cells and by its inability to replicate in PBMCs from a patient with the delta32-ccr5 mutation [29]. The AT2-inactivated viruses were derived from a cell clone of THRO (THRO CL.29) that was produced by co-culturing HEK293T cells transfected with the patient-derived THRO with A66-R5 cells and was identical in amino acid to the parental virus with the exception of Vif T68I, Vpu A8V, and Env R298K mutations. THRO CL.29 productively infected A66 cells expressing CCR5 but did not infect A66 cells that expressed CXCR4 (Julian Bess, personal communication). Following stimulation for 1, 15, and 60-min periods, cells were incubated with cold PBS containing protease and phosphatase inhibitors. Cells were washed, lysed with 2% SDS, and digested enzymatically with trypsin. Phosphopeptides were enriched with titanium dioxide and purified by long gradient UPLC. Enriched samples were analyzed by LC-MS/MS and analyzed with Ingenuity Pathway Analysis (Ingenuity).

### Small GTPase activation assay

Purified CD4+ T cells were resuspended at 5 × 10^6^/ml in RPMI/10% FBS and rested overnight. Cells were then pelleted at 800×*g* for 5 min followed by resuspension in RPMI without FBS, rested at 37 °C for 1 h, and exposed to AT2-inactivated THRO or protein equivalent concentrations of non-viral microvesicles as detailed above. After a 1 min incubation, ice-cold PBS containing protease and phosphatase inhibitors was immediately added, cells were pelleted at 800×*g* for 5 min and resuspended in ice-cold cell lysis buffer. Cells were transferred to a new tube, clarified by centriguation at 10,000×*g* for 1 min at 4 °C and snap-frozen in liquid nitrogen. Following determination of protein concentration, cell lysates were thawed, normalized for protein concentration with lysis buffer, and analyzed using a RhoA, Rac1, and Cdc42 G-LISA activation assay (Cytoskeleton, Inc.) according to the manufacturers instructions.

### Statistics

Data presented as mean values with standard error of the mean unless stated otherwise. All differences with a *p* value of <0.05 were considered statistically significant, correcting for multiple comparisons when appropriate. Statistical analyses were performed using student t-tests within GraphPad Prism v7.0.

## Results

### Viral fusion is not affected by compounds in an epigenetic / post-translational modification screening library

The importance of post-translational modifications (PTMs) on HIV replication has been extensively studied in the context of HIV-1 reactivation from latency, where PTMs regulate the accessibility of host transcription factors and the RNA polymerase machinery to the HIV-1 LTR promoter. Most significantly, the histone deacetylase (HDAC) family of enzymes has become a leading target of pharmacological inhibition in efforts to eliminate viral reservoirs from infected individuals by promoting reactivation of HIV from latency [13]. Recently, we reported this class of drugs has previously unknown effects on early post-entry events in HIV infection, increasing the kinetics and efficiency of reverse transcription and integration [16]. Surprisingly, this enhancement was not due to alterations of histone acetylation but rather to inhibition of the cytoplasmic HDAC6, which regulates tubulin acetylation and microtubule stability [16–19]. To probe for additional epigenetic or post-translational modifications regulating HIV-1 replication, we employed the use of a small molecule inhibitor epigenetic / post-translational modification (PTM) screening library (Additional file 1: Table 1) with our combination reporter virus system that measures both viral fusion and LTR-driven EGFP expression [24]. Viruses bearing a CXCR4-tropic HIV Env were chosen based on the expression of this receptor on a greater percentage of primary CD4+ T cells when compared to the CCR5 co-receptor [30–33]. Compounds within this library have previously been reported to have multiple targets including—but not limited to—histone acetylation and methylation, kinase signaling, and bromodomain and extraterminal domain (BET) protein family members. All compounds screened were prepared in equal experimental conditions and were analyzed across a 3-log concentration to minimize variability of half-maximal inhibitory concentrations (IC50) between compounds. For the initial screen, compounds exhibiting greater than ±0.5 log_2_ fold change (>141.5 or <70.5%) compared to the untreated control in either fusion or infection were flagged as a hit.

**Fig. 1:**
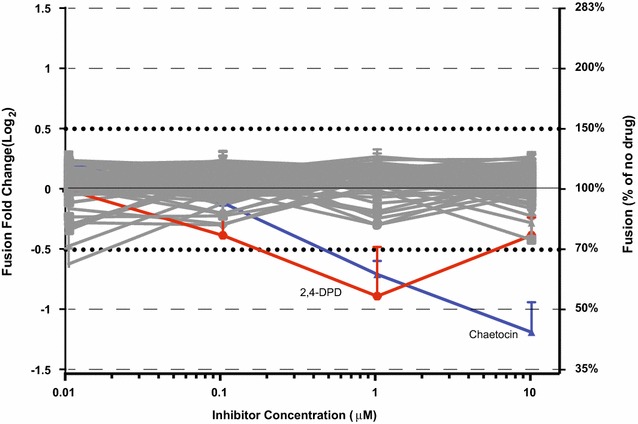
Effect of small molecule inhibitors on viral fusion. Viral fusion was quantified using β-lactamase mediated cleavage of CCF2-AM fluorescent substrate. Primary CD4+ T cells were infected with CXCR4-tropic viruses following treatment to small molecule inhibition. Results are graphed as fold change to untreated control on log_2_ axis. Experimental conditions were performed in triplicate. *Error bars* represent SEM

Our results suggested the majority of compounds tested yielded little to no effect on the level viral fusion when compared to untreated control (Fig. 1). Initially, incubation of primary T cells with the small molecular inhibitors chaetocin and 2,4-DPD suggested an inhibitory effect on viral fusion at high concentrations (Fig. 1). Chaetocin is a fungal metabolite that non-specifically inhibits histone lysine methyltransferases and therefore an effect at the level of viral fusion was unanticipated. However, given that chaetocin contains nitrogen-carbon bonding reminiscent of the fluorescent CCF2-AM dye used in the viral fusion assay, we hypothesized that chaetocin incubation might act as an inhibitor for β-lactamase mediated cleavage of CCF2-AM substrate. To test this, we transfected chaetocin-treated 293T cells with a plasmid encoding for the β-lactamase-Vpr fusion protein and quantified cleavage of CCF2-AM substrate. Incubation with 10 μM chaetocin resulted in a 40% reduction in cleavage of CCF2-AM without affecting cell viability (96.2% of no drug control), suggesting this drug acts not by inhibiting viral fusion but rather by disrupting β-lactamase-mediated cleavage of the CCF2 dye in the assay (data not shown). 2,4-DPD did not inhibit viral fusion in additional experiments (data not shown).

### HDAC, bromodomain, and prolyl hydroxylase inhibitors enhance HIV infection

**Fig. 2:**
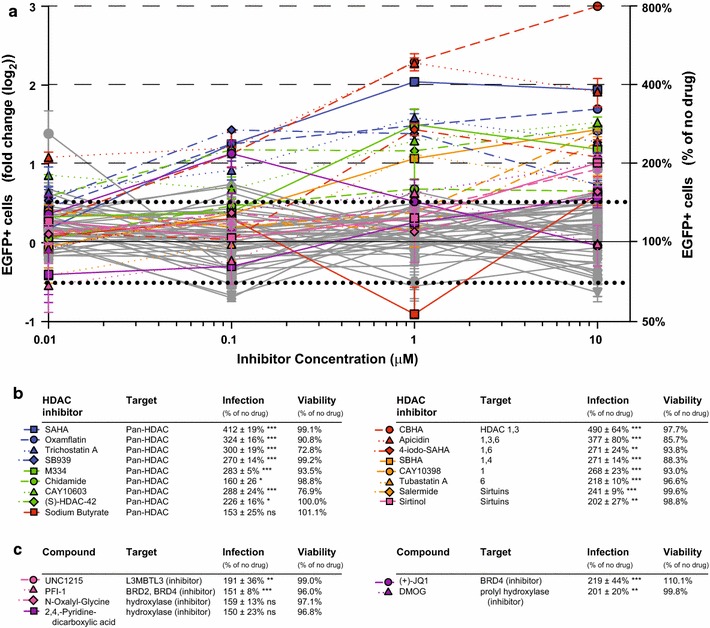
Small molecular inhibitors enhancing viral infection. **a** Analyses of LTR-driven EGFP expression reveal that a large proportion of compounds present in library (49%) have greater than +0.5 log_2_ fold change in viral infection. **b** 17 compounds with greater than +0.5 log_2_ fold-change in EGFP targeted the HDAC family of enzymes, presented with percent infection compared to the no drug control (*ns* not significant, *p < 0.05, **p < 0.01, ***p < 0.001) and with viability data. **c** Six non-HDAC compounds also demonstrated greater than +0.5 log_2_ fold-change in EGFP and targeted the L3MBTL3 methyl-lysine binging protein, cellular hydroxylases, and bromodomain proteins. For both **b** and **c**, the infection and viability data correspond to the drug concentrations with the largest effect on infection with ≥70% viability compared to the no drug control. Compounds in *gray* with greater than +0.5 log_2_ fold change demonstrated cytotoxicity (viability ≤70%)

Examination of viral fusion indicated that treatment with small molecule epigenetic/PTM drugs did not alter HIV entry into CD4+ T cells. Conversely, analyses of LTR-driven EGFP expression revealed dramatic effects with compounds significant increasing cellular susceptibility to HIV-1 infection (Fig. 2a). In support of our previous report [16], 17 hits with greater than +0.5 log_2_ fold change in infection were inhibitors targeting the histone deacetylase class of enzymes (Fig. 2b). These results reinforce our findings that HDAC inhibition increases susceptibility of cells to viral infection [16]. The remaining 6 compounds increasing infection include histone lysine-methyl binding proteins, prolyl hydroxylase inhibitors, and chromatin-associated bromodomain family member BRD2/BRD4 inhibitors (Fig. 2c). BRD2 and BRD4 are of significant interest since inhibitors of these proteins are currently being evaluated as latency reversing agents to eradicate latent HIV reservoirs [34–38]. It will be important to determine whether enhanced infection observed here is the result of known effects, i.e. increasing viral transcription from the LTR promoter, or if—as with the HDAC inhibitors [16]—these compounds have additional mechanisms enhancing infection that could potentially complicate strategies to eradicate latent reservoirs.

### Drugs targeting histone modifying enzymes inhibit HIV infection

**Fig. 3:**
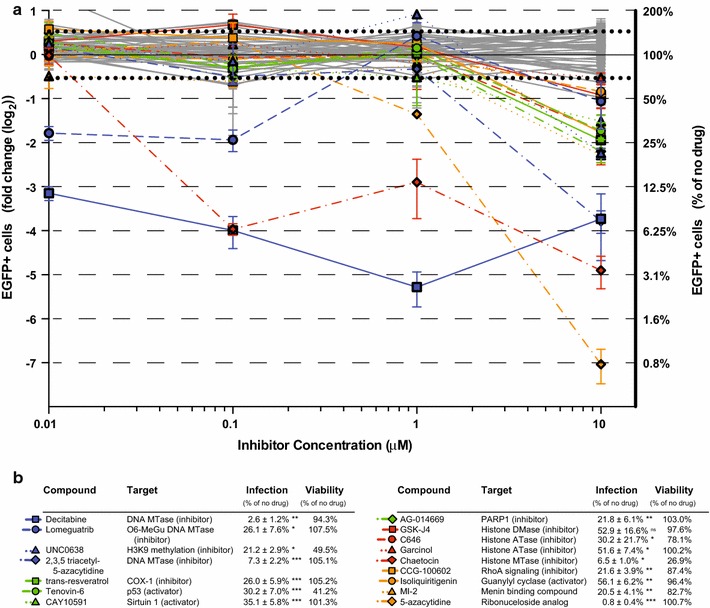
Small molecular compounds reducing viral infection. **a** LTR-driven EGFP expression was reduced by more than −0.5 log_2_ fold change by 16 compounds compared to no drug control (*ns* not significant, *p < 0.05, **p < 0.01, ***p < 0.001). **b** Table listing the 16 compounds reducing HIV infection with target proteins, percent infection, and viability data

The majority of non-HDAC inhibitor hits identified from this screen reduced infection, which could be advantageous in the context of designing strategies to reduce transmission of HIV infection. Compounds with more than −0.5 log_2_ fold-change reduction in EGFP compared to baseline are shown in Fig. 3a, b. Decitabine, 2,3,5-triacetyl-5-azacytidine, and 5-azacytidine are documented nucleoside analog reverse transcriptase inhibitors [39, 40] and strongly reduced infection, providing confirmation that our assay successfully identified known inhibitors of HIV. In addition, several drugs inhibiting histone demethylases, and acetyltransferases reduced HIV infection, including GSK-J4, C646 and garcinol. The finding that multiple regulators of histone modifications influence HIV infection increases confidence that these proteins and pathways play an important role in the HIV replication cycle. In addition, drugs targeting DNA methyltransferases, COX1, PARP1, guanylyl cyclase, and menin binding reduced HIV-1 infection (Fig. 3b). Three drugs—chaetocin, tenovin-6, and UNC0638—demonstrated substantial cytotoxicity 72 h after infection, suggesting that these agents were likely blocking HIV replication by killing cells. These cytotoxic effects were not apparent during analysis of fusion 24 h after infection.

### Validation of compounds with significant effects on infection

**Fig. 4:**
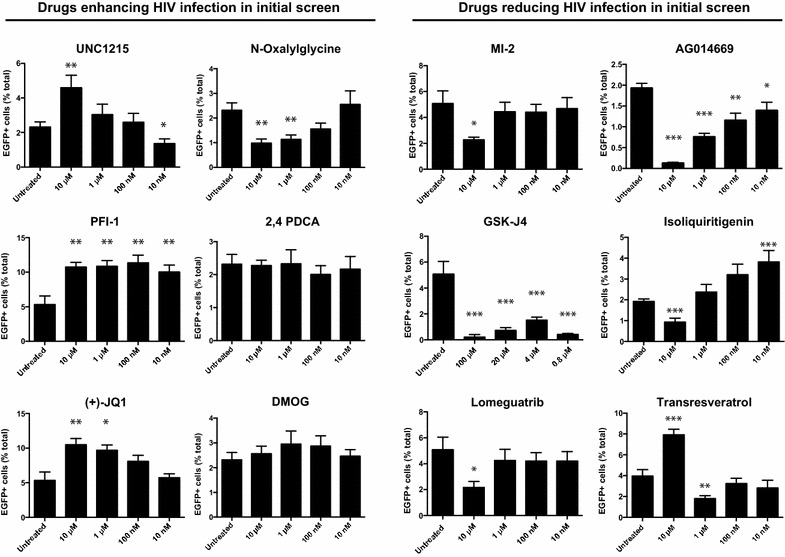
Validation of small molecule inhibitors affecting HIV-1 infection. Significant hits identified from preliminary analysis were validated on a minimum three healthy donors. Compounds having previous effects on HIV-1 infection including nucleoside analogs were excluded from downstream experiments. All conditions were performed in triplicate. *Error bars* represent SEM (*ns* not significant, *p < 0.05, **p < 0.01, ***p < 0.001)

To confirm effects of small molecule inhibitors found to increase LTR-driven EGFP in our infection analyses, we validated compounds on a minimum of three healthy donors (Fig. 4). In some instances, the range of concentrations tested was re-evaluated for compounds with conflicting reports of IC_50_ values (changes are noted in figures when applicable). Validation experiments investigating *N*-oxalylglycine demonstrated a slight inhibitory effect—in contrast to the initial results indicating an increase to infection—while 2,4-PDCA, and DMOG did not affect infection across multiple concentrations (Fig. 4). However, the small molecule inhibitors UNC1215, PFI-1, (+)-JQ1 consistently increased infection across all donors tested. In particular, PFI-1 was highly potent with concentrations as low as 10 nM yielding at a twofold enhancement to viral infection (Fig. 4).

To validate small molecule inhibitors that reduced viral infection, we first eliminated compounds causing cytotoxicity or with previously documented effects on HIV-1 replication including nucleoside analog reverse transcription inhibitors [39, 40]. Our final candidate list included 6 compounds with effects on menin binding, histone modifications, DNA methyltransferases, PARP1, guanylyl cyclase, and COX1: MI-2, GSK-J4, lomeguatrib, AC014669, isoliquiritigenin, and transresveratrol. At the highest concentrations tested, 5/6 compounds exhibited significant reduction in EGFP expression with transresveratrol being the only compound that was not found to reduce EGFP expression in validation experiments (Fig. 4). Of all compounds inhibiting HIV infection, we observed the most potent effects for histone lysine demethylase inhibitor GSK-J4 at all concentrations tested.

### Inhibition of Rho GTPase family members reduces HIV infection

**Fig. 5:**
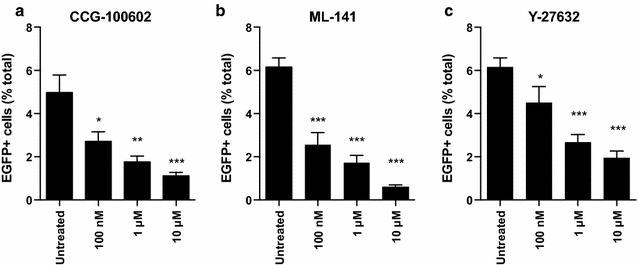
Inhibition of the Rho family GTPases RhoA and Cdc42 and the Rho-associated protein kinase (ROCK) reduce HIV infection. Primary CD4+ T cells were infected with CXCR4-tropic reporter viruses following treatment with small molecule inhibitors targeting **a** Rho A/C signaling, **b** Rho family member Cdc42, and **c** Rho-kinase Rock decrease HIV LTR-driven EGFP expression. *p < 0.05, **p < 0.01, ***p < 0.001. All experimental conditions performed in triplicate. *Error bars* represent SEM

An unexpected observation from our analysis was that treatment with small molecule CCG-100602 reduced LTR-driven EGFP expression in a dose-dependent fashion (Fig. 5a). CCG-100602 is a specific inhibitor of RhoA mediated signaling [25], a pathway that regulates various cytoplasmic and nuclear processes including cytoskeletal dynamics, transcription, and cell cycle progression [41–43]. Given our previous finding that cytoskeletal microtubule networks can regulate the efficiency of HIV infection [16], we chose two additional inhibitors—ML-141 and Y-27632—which target the Rho GTPase Cdc42 and Rho associated protein kinase (ROCK), respectively [44, 45]. In agreement with the RhoA findings, both inhibitors show pronounced reduction in EGFP+ cells suggesting that Rho family GTPases and downstream signaling cascades play a critical role facilitating HIV infection (Fig. 5b, c).

**Fig. 6:**
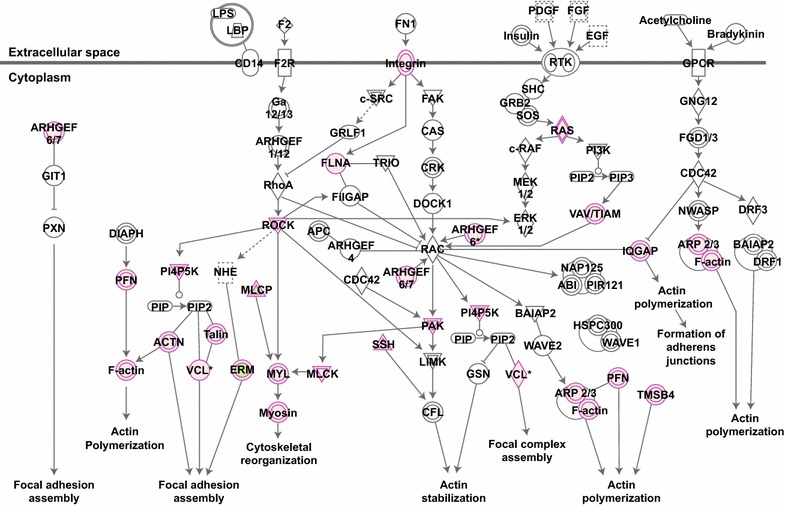
Canonical Rho signaling pathways were identified in a phosphoproteomics screen of HIV-1 signaling via CD4 and CCR5. Phosphoproteomic analysis reveals that numerous Rho family GTPase and ROCK signaling members are dysregulated (*purple*) in response to HIV exposure compared with a similarly prepared microvesicle control. Network analysis was performed using Ingenuity Pathway Analysis software

**Fig. 7:**
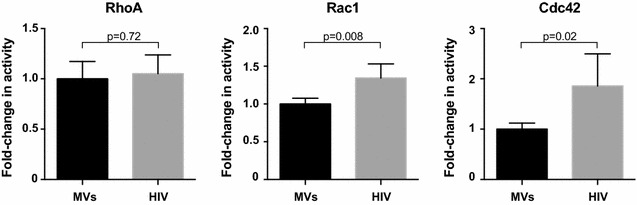
Biochemical GTPase activation assay reveals HIV significantly activates Rac1 and Cdc42. Primary CD4+ T cells were exposed to CCR5-tropic HIV-1 or microvesicles for 1 min and then assessed for RhoA, Rac1, or Cdc42 activation using a small GTPase activation assay (G-LISA). Results were normalized to the microvesicle controls to determine fold-changes in activation. The mean and SD of 6 independent experiments are shown

**Fig. 8:**
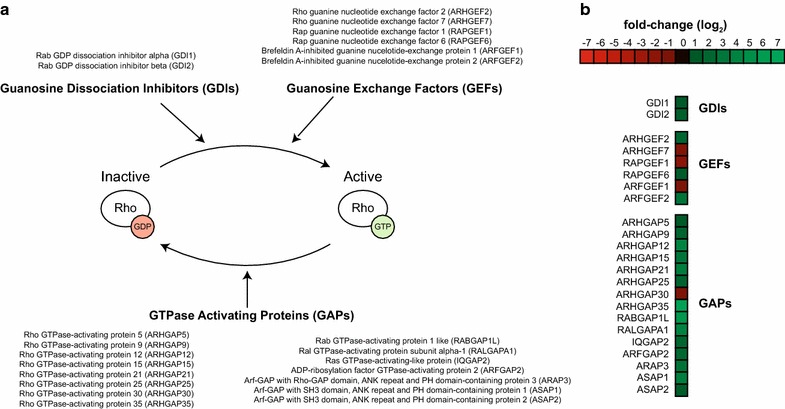
HIV signaling through CD4 and CCR5 alters the landscape of GTPase-regulating proteins. **a** Rho family GTPases are regulated by guanine dissociation inhibitors (GDIs), guanosine exchange factors (GEFs), and GTPase activating proteins (GAPs). GTPase-regulating proteins with ±0.5 log_2_ fold changes in phosphopeptide expression levels following HIV-1 signaling via CD4 and CCR5 as compared to a microvesicle control are listed. **b** Log_2_ fold changes in intensity of phoshopeptides from GTPase-regulating proteins 1 min after exposing cells to inactivated HIV-1 viruses

HIV has also been demonstrated to actively modulate cytoskeletal dynamics to promote infection, including stimulation of acetylated microtubule networks [18] and rearrangements of the cortical actin barrier [46]. To investigate whether HIV signaling regulates Rho family GTPases and downstream kinases, we employed an unbiased phosphoproteomic approach to identify cellular signaling cascades that are dysregulated in response to HIV-1 signaling via CD4 and CCR5. Purified memory CD4+ T cells were isolated from a pooled leukaphersis pack and exposed in parallel to non-viral microvesicles or AT2-inactivated HIV-1 THRO particles. Indeed, Ingenuity Pathway Analysis (IPA) revealed Rho signaling, including proteins involved in the RhoA, Cdc42, and ROCK signaling pathways, as one of the most significantly altered cellular networks in response to HIV-1 exposure (Fig. 6). Importantly, these signaling pathways converge on critical cytoskeletal proteins cofilin, actin, and myosin, and roles for Rho family members in regulating microtubule dynamics and cytoskeletal architecture have been demonstrated [47–50]. To validate that HIV-1 signaling modulates small Rho family GTPases, we performed a biochemical small GTPase activation assay on memory CD4+ T cells from three additional healthy controls. Rac1 and Cdc42 were activity was significantly increased in samples exposed to HIV as opposed to microvesicle controls (*p* = 0.008 and *p* = 0.02, respectively), while RhoA was not significantly altered (*p* = 0.72) (Fig. 7). Activation of Rac1 by HIV signaling has been previously reported [51]; however activation of Cdc42 by HIV-1 signaling in CD4 T cells and its role in promoting infection has not previously been demonstrated. Although RhoA activation not directly observed with either the phosphoproteomics or biochemical assay, phosphorylation of the RhoA substrate ROCK was detected by proteomics. In addition to the proteins mapping to the canonical Rho GTPase signaling pathways in IPA, we also identified more than 20 Rho signaling-associated proteins with significantly different phosphorylation patterns following exposure to inactivated HIV particles, including guanosine disassociation inhibitors (GDIs), guanosine exchange factors (GEFs), and GTPase activating proteins (GAPs) that regulate the activity of Rho family GTPases (Fig. 8a, b). Together, these data suggest that Rho family GTPases RhoA and Cdc42 and the downstream Rho-associated kinase (ROCK) are host dependency factors for HIV replication and that the virus activates Rac1 and Cdc42 and modulates the cellular GTPase landscape via signaling through CD4 and CCR5 on the cell surface.

## Discussion

As an obligate intracellular pathogen, HIV-1 is reliant on interactions with host proteins to facilitate completion of its replication cycle. These proteins, commonly referred to as host dependency factors, have been uncovered through a variety of experimental approaches, most recently high throughput knockdown approaches such as siRNA library screens. While knockdown and knockout studies are effective tools to identify novel host-pathogen interactions, they are not designed to investigate the importance of post-translational modifications in HIV-1 replication.

During previous investigations into the histone deacetylase family of enzymes, we discovered that pharmacological blockade of HDAC6 with specific or pan-HDAC inhibitors significantly enhanced HIV infection [16]. This effect appeared due to the formation of stable acetylated microtubules that serve as conduits for HIV transport to the nucleus, enhancing the kinetics and efficiency of reserve transcription, nuclear import, and integration [16–19]. Overall these data suggest that protein modifications—independent of overall expression level—may play critical roles in HIV replication. In this study, we sought to better define the role for post-translational modifications during HIV replication by utilizing a focused small-molecule inhibitor library with known effects on these mechanisms. This study demonstrates that the use of a low-cost pharmacological library coupled with semi-automated flow cytometry is an effective method to identify novel biological mechanisms modulating HIV infection.

In order to maximize the relevance to in vivo viral replication, experiments were performed using primary CD4+ T cells infected with reporter viruses harboring patient-derived HIV-1 envelope. None of the compounds tested were found to influence viral fusion; however, the fungal metabolite chaetocin inhibited β-lactamase-mediated CCF2 dye cleavage in a fusion-independent manner, likely due to inhibition of the β-lactamase enzyme itself. In stark contrast, we observed 39 compounds within the library that altered HIV infection with greater than ±0.5 log_2_ fold change in EGFP expression. Within this group, 23 were found to increase infection, including 17 separate HDAC inhibitors, greatly strengthening our previous findings that these enzymes impact viral replication [16]. Two additional compounds targeted the BRD2/4 proteins, inhibitors of which are currently being investigated as reactivators of latent HIV proviruses for ‘shock and kill’ strategies to eliminate viral reservoirs [34–38]. Conversely, 15 compounds were found to decrease viral infection, encompassing multiple compounds targeting methylation, acetylation, and cell signaling pathways. Three of these compounds—chaetocin, tenovin-6, and UNC0638—markedly reduced cell viability, suggesting they were inhibiting HIV replication by killing cells. The remaining compounds in the study all demonstrated cell viabilities of at least 70% of the no-drug control.

Although a variety of cellular pathways influencing HIV infection were identified in this screen, several overarching themes were apparent. Multiple compounds tested here targeted mechanisms involved in cellular transcription, including UNC1215, GSK-J4, PFI-1, and (+)-JQ1. UNC1215 and GSK-JR target L3MBTL and JMJD3 enzymes respectively, both of which have reported mechanisms regulating histone methylation and gene silencing [52, 53]. This observation strengthens a role for promoter methylation status in the context of productive versus latent viral gene expression. Investigating the relationship between these compounds and the degree of methylation on HIV-associated DNA may provide further insight into how manipulation of methylation patterns alters HIV infection.

Inhibition of the bromodomain proteins BRD2/BRD4 by compounds PFI-1 and (+)-JQ1 were found to increase infection. BRD family members have become an important focus of shock-and-kill eradication strategies as inhibition has been shown to reverse viral latency by improving Tat interactions at the HIV promoter and by enhancing transcription elongation [34, 35, 54]. Here, we observe that PFI-1 and (+)-JQ1 enhance HIV infection, likely by promoting LTR transcription and thereby disfavoring immediate viral silencing following infection of unstimulated primary CD4+ T cells. However, it will be important to determine if these compounds—like the HDAC inhibitors—have additional mechanisms enhancing infection that could complicate strategies to eradicate latent reservoirs.

The screen performed in this study also revealed an inhibitor of Rho GTPase signaling that decreased HIV infection. The Rho GTPase family members and associated proteins have well-established relationships with cytoskeletal dynamics including actin and microtubules organization. Recently, it has become increasingly clear that the cytoskeleton plays in important role in trafficking the virus from the periphery to the nucleus, increasing the efficiency and kinetics of infection. Building on this initial result, we tested additional inhibitors to the Rho family GTPase Cdc42 and the downstream Rho-associated protein kinase (ROCK), both of which also significantly reduced infection. These results suggest that multiple members of the Rho signaling family are host dependency factors promoting optimal HIV-1 infection of CD4+ T cells. Using a complementary unbiased proteomic approach to identify phosphorylation changes of host proteins following HIV engagement and signaling through CD4 and CCR5, we observed large-scale re-structuring of Rho GTPase signaling pathways including significant phosphorylation changes in more than 20 Rho GTPase associated proteins including the guanosine dissociation inhibitors (GDIs), guanosine exchange factors (GEFs), and GTPase activating proteins (GAPs) that regulate the activity of GTPases. These results were confirmed with a biochemical small GTPase activation assay which confirmed prior results demonstrating that Rac1 is activated by HIV signaling [51] and revealed Cdc42 as be a novel HIV signaling-responsive GTPase.

Exactly how Rho GTPases promote HIV infection remains unclear. Rho family signaling has an extensive list of downstream effectors and has been shown to regulate processes including cytoskeletal dynamics, transcription, endosomal trafficking, cytokinesis, cell cycle, and cell adhesion. The role of Rho family GTPases in the modulation of cellular actin networks is well appreciated. With respect to HIV infection, signaling mediated through ROCK results in phosphorylation of downstream LIM kinases and the actin regulator cofilin, previously implicated in promoting HIV infection of target cells [46]. Specifically, the activation of cofilin by HIV-CXCR4 interactions is thought to assist viral entry by disrupting the cortical actin barrier of CD4+ T cells and facilitating passage into the cytoplasm. Early post-entry viral events are also modulated by actin-interacting proteins such as syntenin-1, ERM, vinculin, WASP, WAVE-2, and Arp2/3, many of which are also directly regulated by Rho family proteins [55]. Furthermore, Rho activation triggers the accumulation of ERM family and vinculin proteins to the plasma membrane to facilitate their involvement in actin remodeling.

More recently, it has become clear Rho family GTPase proteins, including RhoA, Rac, and Cdc42, also play a central role in controlling the dynamic organization and stabilization of microtubules. For instance, RhoA activates the formin protein diaphanous homolog 1 (DIA1, DIAPH1, mDia1), which in turn recruits end binding protein 1 (EB1) and adenomatous polyposis coli (APC), leading to the stabilization of dynamic microtubules [47]. Intriguingly, a role for EB1 in stabilizing microtubules during HIV infection has been reported, mediated by HIV-1 matrix protein interacting with EB1-binding protein Kif4 to promote stable microtubule formation [18]. Our results here indicate that viral manipulation of microtubule networks may in fact originate considerably earlier; namely during engagement of the CD4 and CCR5 receptors. A role for the Cdc42 protein in microtubule stability has also been described, specifically in the reorientation of the microtubule organizing center (MTOC) and microtubules towards the immune synapse in CD4+ T cells and dendritic cells [49, 50]. Finally, the Rho-associated protein kinase (ROCK) has also been implicated as a regulator of microtubule stability via phosphorylation of the tubulin polymerization-promoting protein (TPPP), which in turn interacts with HDAC6 to modulate tubulin acetylation status [48]. Despite these advances in our understanding of Rho GTPase regulation of cytoskeletal dynamics and viral infection, the precise molecular mechanisms through which RhoA, Cdc42, and ROCK promote viral infection remain unclear and are currently under investigation.

## Conclusions

Collectively, this study has reinforced the importance of the cytoskeleton in optimal HIV-1 infection of host cells and implicated RhoA, Cdc42, and ROCK as host dependency factors for viral replication. Furthermore, we have demonstrated that viral engagement of CD4 and CCR5 activates Rac1 and Cdc42 and results in large-scale phosphorylation changes to proteins in the GTPase signaling cascade, indicating the viral hijacking of the cytoskeleton may be initiated during the earliest stages of HIV attachment to a target cell. Importantly, dependence upon Rho family GTPase signaling for optimal infection of target cells is not unique to HIV-1 infection: adenovirus [56], Kaposi’s sarcoma herpesvirus [57, 58], and influenza A virus [59] infections also require intact Rho function, suggesting that manipulation of Rho GTPase signaling pathways to promote cytoskeletal reorganization may be a common feature among many viral infections involving a nuclear stage of the viral life cycle.

## Additional file

**Additional file 1: Table** **1.** Small molecule inhibitors tested with the combination reporter virus system. All compounds were initially tested with an assigned identifier (left column) to remove result bias. Listed half-maximal inhibitory concentrations (IC50) were compiled from previously published studies.
