# Supplementary material for: HIV signaling through CD4 and CCR5 activates Rho family GTPases that are required for optimal infection of primary CD4+ T cells
Source: Retrovirology. 2017 Jan 24;14:4. doi: 10.1186/s12977-017-0328-7 (PMC5259950; doi:10.1186/s12977-017-0328-7)
Supplement: Supplementary file 1 — Additional file 1: Table 1. Small molecule inhibitors tested with the combination reporter virus system. All compounds were initially tested with an assigned identifier (left column) to remove result bias. Listed half-maximal inhibitory concentrations (IC50) were compiled from previously published studies. [file 12977_2017_328_MOESM1_ESM.pdf]

Supplemental Table 1

Epigenetic Screening Library. A. Small molecule inhibitors tested with the combination reporter virus system. All compounds were initially tested with an assigned identifier (left column) to remove result bias. Listed half-maximal inhibitory concentrations (IC_{50}) were compiled from previously published studies.

Identifier	Compound Name	Reported or predicted IC50s
A2	*3-Amino Benzamide*	1.8 µM
A3	*Ellagic Acid*	600-700 nM
A4	*UNC0638*	<15 nM
A5	*Decitabine*	438 nM
A6	*Lomeguatrib*	3 nM
A7	*Tenovin-6*	21 µM (SIRT1) / 10 µM (SIRT2) / 67 µM (SIRT3)
A8	*M 344*	46 nM
A9	*2',3',5'-triacetyl-5-Azacytidine*	200 nM
A10	*trans-Resveratrol*	15 µM (peroxidase) / 3.7 µM (COX-1)
A11	*CAY10591*	20-60 µM
B2	*SB 939*	77 nM
B3	*Suberohydroxamic Acid*	250-300 nM
B4	*Isoliquiritigenin*	1.8 µM
B5	*(+)-JQ1*	18-77 nM
B6	*Daminozide*	500 nM - 2.1 µM
B7	*Sodium Butyrate*	300-400 nM
B8	*Oxamflatin*	15.7 nM
B9	*S-Adenosylhomocysteine*	N/A
B10	*2,4-DPD*	N/A
B11	*EX-527*	98 nM
C2	*PCI 34051*	10 nM
C3	*Apicidin*	15.8 nM - 2 µM
C4	*CCG-100602*	9.8 µM
C5	*(-)-JQ1*	N/A
C6	*GSK-J1 (sodium salt)*	60 nM - 56 µM
C7	*Anacardic Acid*	5-8.5 µM
C8	*Salermide*	20 µM
C9	*UNC0224*	15 nM
C10	*DMOG*	0.1-1 mM
C11	*SAHA*	50 nM
D2	*4-iodo-SAHA*	~ 1 µM
D3	*UNC0321 (trifluoroacetate salt)*	6-9 nM
D4	*CAY10669*	662 µM
D5	*BSI-201*	40-128 µM
D6	*GSK-J2*	>100 µM
D7	*AGK2*	3.5 µM
D8	*Mirin*	66 µM
D9	*Chidamide*	4 µM
D10	*Trichostatin A*	70 nM
D11	*2-PCPA (HCl)*	20.7 µM
E2	*Sirtinol*	38 (SIRT2) / 131 µM (SIRT1)
E3	*(-)-Neplanocin A*	80 nM
E4	*Zebularine*	IC20 = 5-20 µM
E5	*AG-014699*	N/A
E6	*GSK-J4 (HCl)*	>50 µM
E7	*CAY10603*	.002 nM - 7 µM
E8	*Pimelic Diphenylamide 106*	150 nM - 5 µM (HDAC 1) / 180 µM (HDAC II)
E9	*3-Deazaneplanocin A*	0.2-1 µM
E10	*CAY10398*	10 µM
E11	*1-Naphthoic Acid*	N/A
F2	*C646*	1.6 µM
F3	*Cl-Amidine*	5.9 µM
F4	*Delphinidin Chloride*	30 µM
F5	*IOX1*	1.7-20.5 µM
F6	*GSK-J5 (HCl)*	>100 µM
F7	*Chaetocin*	0.8-3 µM
F8	*(S)-HDAC-42*	N/A
F9	*N-Oxalylglycine*	2.1-5.6 µM (PHD1/2)
F10	*2,4-Pyridinedicarboxylic Acid*	1.5 µM
F11	*Nicotinamide*	N/A
G2	*Tubastatin A (trifluoroacetate Salt)*	15 nM (HDAC6)
G3	*F-Amidine (trifluoroacetate Salt)*	21.6 µM
G4	*PFI-1*	98 nM (BRD2) / 0.22 µM (BRD4)
G5	*MI-2 (HCl)*	0.45 µM
G6	*Valproic Acid (Sodium Salt)*	2 mM
G7	*Splitomicin*	60 µM (SIR2p)
G8	*MS-275*	300 nM (HDAC-1)
G9	*AMI-1 (sodium salt)*	3-8.8 µM
G10	*CAY10433*	30 µM
G11	*Sinefungin*	0.1-20 µM
H2	*Garcinol*	7 µM (p300) / 5 µM (PCAF)
H3	*JBG1741*	N/A
H4	*5-Azacytidine*	.2µM
H5	*MI-negative control(HCl)*	193 µM
H6	*Tenovin-1*	10µM
H7	*CBHA*	10-7 nM
H8	*RG-108*	115 nM
H9	*UNC1215*	40 nM
H10	*Piceatannol*	25 µM
H11	*Suramin (sodium salt)*	4.9 µM
